# No observed effect of homologous recombination on influenza C virus evolution

**DOI:** 10.1186/1743-422X-7-227

**Published:** 2010-09-14

**Authors:** Guan-Zhu Han, Maciej F Boni, Si-Shen Li

**Affiliations:** 1 State Key Laboratory of Crop Biology, College of Agronomy, Shandong Agricultural University, Tai'an, Shandong 271018, China; 2 Oxford University Clinical Research Unit, Ho Chi Minh City, Vietnam; 3 MRC Centre for Genomics and Global Health, University of Oxford, Oxford, UK; 4 Current Address: Department of Ecology and Evolutionary Biology, University of Arizona, Tucson, Arizona 85721, USA

## Abstract

The occurrence of homologous recombination in influenza viruses has been under some debate recently. To determine the extent of homologous recombination in influenza C virus, recombination analyses of all available gene sequences of influenza C virus were carried out. No recombination signal was found. With the previous evidence in influenza A and B viruses, it seems that homologous recombination has minimal or no effect on influenza virus evolution.

## Background

The influenza C virus belongs to the *Orthomyxoviridae *family and is a common cause of mild upper respiratory tract illness. Seroepidemiological studies indicate that it is widely distributed around the world, but isolated infrequently, and the majority of humans acquire antibodies to the virus early in life [1]. In contrast with influenza A and B viruses, the influenza C virus genome consists of only seven single stranded negative-sense RNA segments, the PB2, PB1, P3, HE, NP, M, and NS segments. Analysis of the full genome sequence of type C influenza viruses suggested that reassortment between two different type C influenza viruses occurs frequently in nature [2,3]. Furthermore, influenza C virus has been suggested to be involved in heterologous RNA recombination events [4].

Largely because their RNA is always encapsidated by a ribonucleoprotein complex (RNP), single-stranded negative-sense RNA viruses are generally believed to undergo a low rate of homologous recombination [5]. However, there is increasing evidence of homologous recombination involving negative-strand RNA viruses like Newcastle disease virus [5-8], Zaire Ebola virus [9], measles virus [10], and canine distemper virus [11,12]. Homologous recombination has also been demonstrated in the laboratory for respiratory syncytial virus and hantavirus [13,14]. However, the evidence for homologous recombination in influenza viruses has been sparse and controversial. Gibbs et al. proposed that homologous recombination had occurred in the HA gene of 1918 Spanish flu virus [15]. However, the apparent recombination event described by Gibbs et al. is much more likely the result of a difference in the substitution rate between HA1 and HA2 [16]. Several recent studies provide some new evidence for recombination in influenza A virus [17-19]. In particular, He et al. provide evidence for a clade of three recombinant avian influenza sequences [17], but large-scale analyses have shown that anomalies in the influenza sequence database, possibly caused by sample contamination, may generate false-positive recombination signals [20-22]. Indeed, when controlling for sequence quality, the evidence for homologous recombination is weak in both influenza A and B viruses [20,22,23]. Given the evidence to date, homologous recombination seems to play little or no role in the evolution of influenza A and B viruses. In a previous small scale analysis, patterns of sequence variation compatible with the action of recombination, but not definitive evidence, were observed in influenza C virus [5]. The increasing availability of genome sequences of influenza C virus may have the potential to shed new light on the role of homologous recombination in the evolution of influenza C virus.

## Results and Discussion

To determine the role of homologous recombination in the evolution of influenza C virus, we gathered all 722 publicly available influenza C virus sequences representing all seven RNA segments on April 10, 2010 (Table 1) and performed recombination analyses as described elsewhere [20]. Briefly, the sequences were obtained from the Influenza Virus Resource [24] and then aligned using MUSLE v3.6 [25]. All sequence alignments are available from the authors upon request. Recombination signals were tested using the 3SEQ algorithm [26]. 3SEQ tests all possible triplets in a data set for a mosaic recombination signal using a nonparametric statistic for mosaicism, and it reports p-values and breakpoint ranges. 3SEQ's nonparametric Δ-statistic is a special type of Mann-Whitney U-test on binary outcomes where, rather than identifying one outcome coming sequentially before another outcome, one outcome is identified to cluster in the middle of the sequence of the other outcomes. For example, both the Mann-Whitney U-test and 3SEQ's Δ-statistic would identify 000010000111101111 as a statistically significant pattern where the 1 s appear sequentially after the 0s; however, only the Δ-statistic would identify 000111011101100000 as a statistically significant sequence where the 1 s cluster in the middle of a sequence of 0 s. This latter sequence of zeros and ones is the type of pattern that must be detected with recombination software that identifies mosaic signals.

**Table 1:** Summary of results in this study

Segment	No. of sequences	No. of distinct sequences	Alignment length (nt)	Min Bruen Φ p-values (window sizes = 50,100,150)
PB2	90	55	2365	0.43
PB1	88	49	2363	0.91
P3	88	33	2183	0.24
HE	138	116	2075	0.74
NP	87	61	1809	0.86
MP	108	79	1180	0.50
NS	123	94	935	0.36

1. All p-values by 3SEQ were one.

2. No. of recombination signals by Chimera, GENECONV and RDP were zero.

3SEQ is among the most powerful recombination detection methods, especially in datasets with high nucleotide diversity [26]. Mean pairwise distances for the influenza C data sets analyzed here ranged from 7nt to 75nt, and it is possible that for this range of diversity 3SEQ would not have enough power to detect recombination. To determine if recombination could not be found because of the low diversity of influenza C viruses, we tested all seven segments with a homoplasy method (Bruen's Φ [27]) as homoplasy methods are intended to detect recombination in data sets with low levels of diversity. Window sizes for this methods were set to 50, 100, and 150, and no recombination signal was found, as all p-values were greater than 0.24 (Table 1). In addition, recombination detection was also performed by using Chimaera, GENECONV, and RDP, which are available in the RDP (Recombination Detection Program) software package [28]. None of the seven influenza C virus data sets analyzed here contained sequences with statistically significant recombination signals (Table 1).

In this study, we used all influenza C viral sequences available in Influenza Virus Resource. Although the majority of segments PB1, PB2, P3, and NP sequences are incomplete, their lengths are enough to find signals if recombination did occur frequently [27]; if recombination occurred infrequently, then the low levels of nucleotide diversity for some influenza C segments may make it difficult to detect recombination signals.

Influenza viruses evolve through a variety of genetic mechanisms, rapid mutation, frequent reassortment and rarely non-homologous recombination [29]. Homologous recombination has been proposed as an important evolutionary force for evolution of influenza A virus [17,18], but the evidence to date has been weak and few studies have carried out quality-controlled experiments to determine whether homologous recombination is present or extensive in influenza virus evolution [22]. Here we demonstrate that, given the limited sequence diversity in current sequence data, there are no observed homologous recombination signals for influenza C viruses.

## Conclusion

In our study, no homologous recombination signal was found in influenza C virus. Given the present evidence, homologous recombination, if it exists, may only play a minor role in the evolution of influenza virus.

## Competing interests

The authors declare that they have no competing interests.

## Authors' contributions

GZH, SSL designed the study; GZH, MFB performed the research; GZH drafted the manuscript. MFB, SSL revised the manuscript. All authors read and approved the final manuscript.
